# Reducing the Damage of Ox-LDL/LOX-1 Pathway to Vascular Endothelial Barrier Can Inhibit Atherosclerosis

**DOI:** 10.1155/2022/7541411

**Published:** 2022-03-29

**Authors:** Xiaopeng Guo, Yishan Guo, Zhiwen Wang, Bingxin Cao, Chuansheng Zheng, Zhuanglin Zeng, Yumiao Wei

**Affiliations:** ^1^Department of Radiology, Union Hospital, Tongji Medical College, Huazhong University of Science and Technology, Wuhan 430022, China; ^2^Department of Cardiology, Union Hospital, Tongji Medical College, Huazhong University of Science and Technology, Wuhan 430022, China; ^3^Department of Cardiology, Binzhou Medical University Hospital, Binzhou 256600, China; ^4^Department of Emergency Medicine, Union Hospital, Tongji Medical College, Huazhong University of Science and Technology, Wuhan 430022, China

## Abstract

**Aim:**

The destruction of the vascular endothelial barrier mediated by Ox-LDL is the initial link to atherosclerosis. Here, we aimed to determine whether the immunological intervention with Ox-ApoB polypeptide fragment (Ox-ApoB-PF) can block the deposition of Ox-LDL in vascular endothelial cells through LOX-1 receptors, thereby protecting the barrier function and survival status of vascular endothelial cells and inhibiting the progression of atherosclerosis.

**Methods and Results:**

In order to determine the harm of Ox-LDL to vascular endothelial cells and the protective effect of immune intervention with Ox-ApoB-PF, we conducted a series of corresponding experiments in vitro and in vivo. The in vitro results showed that Ox-LDL can activate endothelial cell apoptosis pathway; reduce the expression of endothelial junction proteins; affect the migration, deformation, and forming ability; and ultimately destroy the vascular endothelial barrier function. The increased permeability of endothelial cells led to a sharp increase in the phagocytosis of Ox-LDL by macrophages under the endothelial layer. Meanwhile, Ox-LDL stimulation induced a significant upregulation of LOX-1 in endothelial cells and increased the expression of endothelial cell chemokines and adhesion factors. Ox-ApoB-PF antibodies can significantly reduce the abovementioned harmful effects. The in vivo results showed that active immune intervention through Ox-ApoB-PF can protect the endothelial barrier function; reduce macrophage deposition and the inflammatory response in plaques; alleviate lipid deposition in the plaques, as well as apoptosis and necrosis; and increase the ability of liver macrophages to clear Ox-LDL. Eventually, the progression of plaque and the formation of necrotic cores in plaques can be inhibited.

**Conclusions:**

An Ox-ApoB-PF antibody may protect the endothelial cell physiological function and survival status by blocking the combination of Ox-LDL/LOX-1 in vascular endothelial cells. Immune intervention with Ox-ApoB-PF inhibits the occurrence and development of atherosclerotic lesions by protecting the vascular endothelial barrier function.

## 1. Introduction

Cardiovascular death is one of the leading causes of death in our country. Approximately 40% of deaths each year are caused by cardiovascular events, and atherosclerosis is the leading cause of cardiovascular events [1, 2]. Previous studies have shown that atherosclerosis is a disease caused by lipid metabolism disorders and is characterized by chronic inflammation and an abnormal immune response [3]. Additionally, the deposition of oxidized lipids under the blood vessel wall and the destruction of the endothelial cell connection barrier are considered to be the most critical factors for the initiation of atherosclerosis [4, 5]. According to previous studies, oxidized low-density lipoprotein and macrophages are the main pathogenic factors for the development of atherosclerosis [6, 7]. Under proatherogenic conditions, circulating LDL is easily oxidized to Ox-LDL by oxygen free radicals. The specific epitope on the surface of the latter allows inflammation-related phagocytes, vascular endothelial cells and smooth muscle cells to recognize, bind, and phagocytose Ox-LDL through their inherent pattern recognition receptors [8]. Generally, Ox-LDL triggers a series of inflammatory signalling pathways after being engulfed, such as c-Jun NH2-terminal protein kinase (JNK), which leads to the expression and release of a large number of inflammatory factors and endothelial adhesion factors [9–11]. Meanwhile, Ox-LDL that is deposited on the vascular endothelium is not only phagocytosed by vascular endothelial cells but also further phagocytosed by macrophages under the endothelium through scavenger receptors on the surface of macrophages, such as CD36 [12]. Macrophages engulf Ox-LDL but cannot degrade Ox-LDL, which eventually leads to the foaming of macrophages to form foam cells. Over time, the core part (which is necrotic) in atherosclerotic plaques is formed, which intensifies the progression of atherosclerosis and even causes plaque rupture, leading to cardiovascular death [13]. Therefore, Ox-LDL is the most important harmful factor in the occurrence and development of atherosclerosis, and blocking its phagocytosis by vascular endothelial cells has potential as a therapeutic strategy.

Under normal physiological conditions, vascular endothelial cells are connected to each other to form a vascular endothelial barrier to maintain vascular homeostasis, and this barrier prevents circulating cells and proteins from infiltrating the tissue from the vascular lumen [14]. However, the barrier is easily destroyed. Even a slight amount of inflammation can cause the destruction of the barrier function and cause a higher permeability, triggering the invasion of macrophages and neutrophils into the circulation and activating a variety of inflammatory pathways in the blood vessel wall to cause atherosclerosis [15]. Studies have shown that with the destruction of the endothelial barrier function, the deposited Ox-LDL induces the high permeability of the vascular endothelial monolayer cells by affecting the expression of cell tight junction proteins (such as ZO-1 and VE-cadherin) [16].

Lectin-like oxidized low-density lipoprotein receptor-1 (LOX-1), which is mainly expressed in endothelial cells, belongs to the class E scavenger receptor family. It is the most important receptor that recognizes Ox-LDL in the endothelial cells and phagocytose it which can induce lipid stress to damage the endothelial barrier function. At the same time, the activation of LOX-1 can mediate the production of reactive oxygen species and NLRP3 inflammasomes and induce pyrolysis of endothelial cells. In addition, LOX-1 also causes endothelial cell apoptosis through the apoptosis pathways Bax/Bcl-2, caspase-3, caspase-8, and caspase-9. However, the further infiltration of lipids, the phagocytosis and clearance of apoptotic endothelial cells by inflammatory cells, and the susceptibility to thrombosis caused by the loss of endothelial cells all significantly contribute to the progression of atherosclerosis and concurrent coronary events. Early studies have shown that the use of LOX-1 blocking antibodies or a gene knockout of LOX-1 can benefit the survival of the vascular endothelial barrier caused by Ox-LDL [17]. However, the implementation of the two methods is very inconvenient and uncontrollable. Therefore, it will be of great significance to find more feasible measures to intervene in this mechanism.

ApoB is the main component of low-density lipoprotein, and its main function is to transport lipids. The core pathogenic component of Ox-LDL is the change of oxidized ApoB to a specific epitope of the oxidation reaction, which becomes a damage-related molecular pattern and is phagocytosed and deposited by vascular endothelial cells and macrophages that have infiltrated under the endothelium through their pattern recognition receptors. Previous studies have shown that a vaccine derived from ApoB-100 exerts a therapeutic effect by inhibiting atherosclerosis, and a P210 vaccine can reduce the progression of atherosclerosis in ApoE^−/−^ mice by approximately 40% [18, 19]. These findings indicate that the vaccine against ApoB-100 is a potential therapeutic agent for the treatment of atherosclerosis, but the specific inhibitory mechanism remains unclear. Therefore, we envisaged that by using an Ox-ApoB-PF. Containing the adhesion and aggregation sites of endothelial proteoglycans to prepare the corresponding immune preparations and then intervening in the early stage of atherosclerosis, the binding of Ox-LDL and LOX-1 could be blocked to protect the endothelial barrier connection, reduce vascular inflammatory stress, and inhibit atherosclerosis.

## 2. Materials and Methods

### 2.1. Preparation of Ox-ApoB-PF and Ox-ApoB-PF Antibody

Antibodies against Ox-ApoB and Ox-ApoB were prepared as described previously [20, 21]. Peptides containing amino acids 3136–3155 of ApoB-100, modified with malondialdehyde (MDA), were synthesized using a peptide synthesizer (PSSM-8, Shimadzu, Japan) [19]. The purity was determined to be 95% by high-performance liquid chromatography. In order to get the Ox-ApoB antibody, Japanese white rabbits were immunized with Ox-ApoB for 9 weeks. After that, blood was collected from these rabbits to determine the Ox-ApoB antibody titer by enzyme-linked immunosorbent assay (ELISA). The immunized rabbits with a high titer (>1 : 12000) were anaesthetized and sacrificed by exsanguination from the common carotid arteries. We purify the IgG antibody by affinity chromatography and determine the affinity of the antibody. The antibody titer level reaches above 1 mg/ml and is used for in vitro research.

### 2.2. Cell Culture

HUVECs were cultured in endothelial cell medium (ECM) containing 10% fetal bovine serum (FBS). THP-1 monocytes were cultured in the RPMI 1640 medium containing 10% fetal bovine serum. THP-1 cells with good growth status were chosen and incubated with PMA for 24–48 h to differentiate into macrophages with a phagocytic function. All cells were cultured at 37°C with 5% CO_2_.

### 2.3. Determination of Cell Viability

Cell counting kit-8 was used to determine the activity of cells. Before the following experiment, the cytotoxicity of the antibody to the Ox-ApoB on HUVECs was tested. HUVECs were first cultured in 96-well plates and then intervened with different concentrations of specific antibodies for 24 h. After that, CCK-8 solution was added to each well and incubated at 37°C for 1 h. Finally, the optical density (OD) values at 450 mm were measured by the microplate reader.

### 2.4. Western Blot

After the cells were treated, they were lysed with RIPA (AntGene, ANT060) lysis buffer to extract the total protein in the cells. Then, the protein concentration was determined by the BCA protein detection kit, and a 10% SDS-PAGE gel was prepared for electrophoresis. After electrophoresis was completed, the proteins were transferred to a polyvinylidene fluoride membrane. A 5% skimmed milk solution was prepared and it was sealed with milk for 2 hours after the transfer was completed. Anti-LOX-1 (Abcam, ab203246), anti-MCP-1(NOVUS, NBP2-22115), anti-VCAM-1(GeneTex, GTX110684), anti-ZO-1(Cell Signalling Technology,13663S), anti-VE-cadherin (Cell Signalling Technology, 2500S), and anti-GAPDH (AntGene, ANT012) antibodies were incubated overnight at 4°C. A rabbit HRP conjugate was used as the secondary antibody. The optical density of the Western blot was quantified by using Image Lab software.

### 2.5. Immunofluorescence

Cellular immunofluorescence was used to detect the expression of VE-cadherin (Cell Signalling Technology, 2500S) and ZO-1(Cell Signalling Technology, 13663S) in the cells. After washing the cell slides of the different treatment groups with PBS, they were fixed with 4% paraformaldehyde for 10 min. Then, the cells were blocked with 10% goat serum for 30 min. An appropriate amount of the corresponding primary antibody diluted with PBS was added, and they were placed in an incubator at 4°C overnight. The slides were warmed to room temperature for 1 hour and were washed with PBS, fluorescent-labelled secondary antibody was added, and the slides were incubated at room temperature for 1 hour. The nuclei were washed with PBS, and DAPI was used to counterstain the nuclei for 5 minutes. They were then evaluated under a confocal laser microscope for observation.

### 2.6. Transwell

Transwell migration experiments were used to detect the effect of Ox-LDL on the migration and deformation ability of HUVECs and to observe the protective effect of the antibodies. Cells were pretreated with 50 *μ*g/ml Ox-LDL alone or in combination with 100 ng/ml of antibody for 24 hours to obtain a cell suspension. A Transwell chamber was chosen that had an 8 *μ*m pore size, 200 *μ*l cell suspension was added to the upper layer, and 500 *μ*l medium containing 10% or 20% FBS was added to the lower layer. After 24 hours, the cells were removed, and the cells in the upper layer of the cell were scraped off with a cotton swab. After washing with PBS, the cells were fixed with 4% paraformaldehyde for 10 min. Then, the cells were placed upside down on filter paper and dried after washing with PBS. The cells were stained with 0.1% crystal violet dye solution for 10 min and were washed with ddH_2_O. After the slides were placed under an optical microscope, 5 fields of view were randomly selected to take pictures and count cells at a 100x magnification.

### 2.7. Cell Scratch Wound Healing Assay

The cell scratch wound healing assay was used to detect the effect of Ox-LDL on the migration of HUVECs and to observe the protective effect of the antibody. HUVECs were cultured in a 12-well plate. When the cell density reached approximately 90%, a 200 *μ*l pipette tip was used to make a scratch, and different stimulations were given according to the different groups. Photomicrographs were taken after 0 h, 12 h, and 24 h. Image-Pro Plus software was used to measure the change in the scratch area over time.

### 2.8. Tube Formation Assay

The tube formation assay was used to detect the effect of Ox-LDL on the angiogenesis ability of HUVECs, and the protective effect of the antibody was evaluated. HUVECs were seeded onto 48-well plates, which were precoated with Matrigel matrix (150 *μ*l/per well) and complete medium (300 *μ*l/per well) for 12 hours. Then, the tube formation was photographed at 6 h and 9 h, and the total tube length in 5 fields was quantified using Image-Pro Plus software.

### 2.9. Animals

Seven-week-old male ApoE^−/−^ mice were used in this study (Beijing HFK Bioscience Ltd., China). The mice were randomly divided into three groups: the control group, the F-adjuvant group, and the immunization group. Mice were housed in a temperature-controlled environment (25°C ± 2°C) with a 12 h light/dark cycle and had free access to food and water.

The immune intervention and a high-fat diet (HFD) were carried out at the same time. The immune intervention was carried out at 1, 4, 7, and 10 weeks after the HFD. The mice were sacrificed after 12 weeks of being fed the HFD, and samples were obtained. The synthesized ApoB polypeptide fragment oxidized by MDA was coupled to the carrier protein keyhole limpet hemocyanin, followed by emulsification with an equal volume of Freund's adjuvant. Then, the experimental animals were immunized. During the experiment, the mice in the immunization group received multiple subcutaneous injections of 200 *μ*g of Ox-ApoB-PF every three weeks. The control group was injected with the same dose of adjuvant.

### 2.10. Atherosclerotic Lesion Analysis

After the heart was perfused, the entire aorta was removed from the root of the aorta to the bifurcation in the area of the iliac bone, and the fatty tissue around the blood vessel was carefully removed under a microscope and was opened longitudinally. Subsequently, the aorta was fixed, was stained with ORO, and was photographed with a digital camera (Nikon, Japan). Image-Pro Plus software was used to measure the total surface area and the ORO-positive lesion area. The percentage of the surface area that had ORO-positive areas was assessed.

### 2.11. Histology and Immunofluorescence Staining

The aortic valve annulus (6 *μ*m thickness) of the heart was stained with ORO and H&E staining. Then, the slices were observed under an optical microscope. For immunofluorescence staining, the aortic valve ring sections were incubated with antibodies against MCP-1 (NOVUS, NBP2-22115), VCAM-1 (Abcam, ab134047), ZO-1 (Abcam, ab221547), CD31 (GeneTex, GTX20218), TUNEL (Servicebio, G1501), and LOX-1(Abcam, ab203246). Immunofluorescence staining of the liver was performed by incubating a tissue section (6 *μ*m thick) of the liver with antibodies against F4/80(Cell Signalling Technology, 70076) and ABCA1 (ABclonal, A7228). The immune complexes were detected by fluorescently labelled secondary antibodies, and DAPI was used to stain the nuclei. Then, the slices were observed with a fluorescence microscope, and Image-Pro Plus was used for the corresponding statistical analysis.

### 2.12. Statistical Analysis

Data were statistically analyzed and graphed using GraphPad Prism 5 (GraphPad Software, USA). All results are presented as the mean values  ±  standard deviations. Statistically significant differences between the groups were determined by Student's *t*-test. Multiple comparisons were made among ≥3 groups using a one-way ANOVA followed by the Bonferroni post hoc test. ^∗^*P* < 0.05, ^∗∗^*P* < 0.01, ^#^*P* < 0.05, and ^##^*P* < 0.01 were considered statistically significant.

## 3. Results

### 3.1. The Transwell Coincubation Model Confirmed That the Antibodies Can Reduce Lipid Deposition under Endothelial Cells and in the Inner Membrane

**Figure 1:**
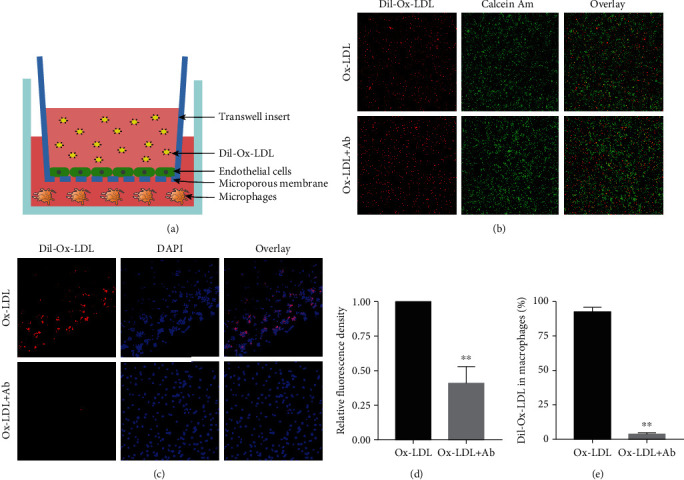
The Transwell coincubation model confirmed that antibodies can reduce lipid deposition under endothelial cells and in the inner membrane. (a) Schematic diagram of the Transwell coincubation model. (b) Comparison of endothelial cells phagocytosing Dil-Ox-LDL under confocal laser irradiation in the control group and the antibody intervention group (100x). (c) Comparison of macrophages in the control group and the antibody intervention group phagocytosing Dil-Ox-LDL under confocal laser irradiation (40x). (d) Taking the fluorescence density of Dil-Ox-LDL phagocytosed by endothelial cells in the control group as baseline 1, the percentage of red fluorescence density of Dil-Ox-LDL phagocytosed by endothelial cells in the antibody intervention group. (e) Taking the fluorescence density of Dil-Ox-LDL phagocytosed by macrophages in the control group as the baseline 100%, the percentage of Dil-Ox-LDL phagocytosed by macrophages in the antibody intervention group. Values are expressed as mean ± SD. Student's *t*-test was applied for determining the significance of data. ^∗∗^*P* < 0.01.

First, we designed a Transwell coincubation model to simulate endothelial and subintimal macrophages to study the lipid infiltration of endothelial cells, the damage of the endothelial barrier, the infiltration of Ox-LDL into the subendothelial area, and whether the specific antibody can alleviate the effect. The upper layer of the chamber (0.4 *μ*m pore size) was tightly inoculated with endothelial cells, and the lower layer was evenly inoculated with human THP-1-derived macrophages. After adding Dil-Ox-LDL, the corresponding detection was carried out (Figure 1(a)). Under laser confocal microscopy, we found that Ox-LDL labelled with the red fluorescent probe Dil could be engulfed by endothelial cells (calcein Am staining) and was deposited in the cytoplasm, and an intervention with a specific antibody significantly reduced the deposition in the endothelial cells (Figures 1(b) and 1(d)). Similarly, we observed the phagocytosis of macrophages located under the inner membrane by using a confocal laser and found that Dil-Ox-LDL penetrates the endothelial cell gap and is engulfed by macrophages in the lower inner membrane. After intervention with specific antibodies, the phagocytosis of Ox-LDL by macrophages under the inner membrane was significantly reduced (Figures 1(c) and 1(e)). The above results indicate that Ox-LDL can be engulfed by endothelial cells under normal circumstances, can enter the subendothelium through the endothelial junction, and can be engulfed by macrophages, triggering a series of harmful reactions. Interventions with specific antibodies can significantly inhibit the deposition of Ox-LDL in the endothelial cells.

Specific antibodies have no cellular toxicity and can reduce the damage by Ox-LDL in endothelial cell migration.

**Figure 2:**
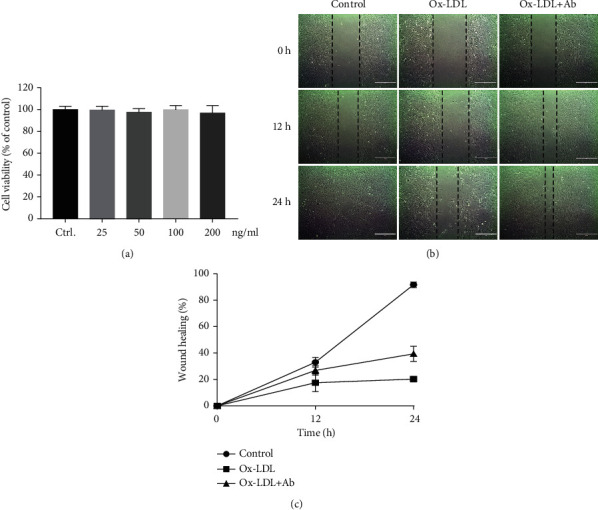
Specific antibodies have no cellular toxicity and can reduce the damage by Ox-LDL in endothelial cell migration. (a) Effects of the specific antibodies on cell viability of HUVECs. (b) Comparison chart of the scratch results in the three groups at 0 h, 12 h, and 24 h. (c) Trend statistics of the percentage of scratch healing in the three groups within 24 hours. Values are expressed as mean ± SD.

To determine the cellular toxicity of the specific antibodies, we carried out the related experiment. From the result of CCK-8, we found that there was no significant change in cell viability with increasing antibody concentrations (Figures 2(a)). This data indicated that the specific antibodies have no cellular toxicity on HUVECs. To further verify the damage of Ox-LDL to endothelial cells and the effect of specific antibodies, we designed a scratch experiment to prove whether Ox-LDL inhibits the migration ability of endothelial cells and whether the intervention with an antibody can reduce the harm of Ox-LDL. The results proved that after 24 h of observation, Ox-LDL stimulation indeed greatly inhibited the migration ability of endothelial cells. Compared with the control group (wound healing 91.7 ± 2.1%), the subendothelial scratches in the Ox-LDL group basically did not heal (wound healing 20.3 ± 2.1%) at 24 h. However, after intervention with the antibody, the scratch healing of endothelial cells (wound healing 39.4 ± 5.7%) was significantly different from that of the Ox-LDL group (Figures 2(b) and 2(c)). These data show that Ox-LDL has a great destructive effect on the migration of endothelial cells, and intervention with an antibody can greatly reduce this damage.

### 3.2. Specific Antibody Intervention Can Significantly Reduce the Damage by Ox-LDL in Endothelial Cell Tube Formation and Migration Ability

**Figure 3:**
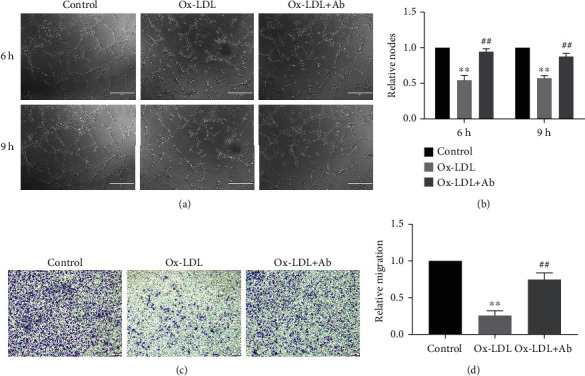
Specific antibody intervention can significantly reduce the damage by Ox-LDL in endothelial cell tube formation and migration ability. (a) Comparison of the results of the three groups at 6 h and 9 h. (b) A statistical chart of the relative ratio of the three components of the tube. The control group result was set as 1. Values are expressed as mean ± SD. ^∗∗^*P* < 0.01 vs. the control group, ^##^*P* < 0.01 vs. the Ox-LDL group. (c) Comparison chart of three groups of Transwell results. (d) A statistical chart of the relative ratios of the three groups of migration. The control group result was set as 1. Values are expressed as mean ± SD. ^∗∗^*P* < 0.01 vs. the control group, ^##^*P* < 0.01 vs. the Ox-LDL group.

In addition, we also tested the damage caused by Ox-LDL in the endothelial cell formation and migration ability by tube formation experiments and Transwell experiments, and we evaluated whether the antibody intervention can inhibit the damage. The tube formation experiment was used to observe the three groups at 6 h and 9 h and found that the Ox-LDL stimulation group was significantly different compared with the control group (set as 1), the tube formation ability was significantly weakened (0.54, 0.57), and there was almost no complete tubular structure. However, in the antibody intervention group, the tube-forming ability of the endothelial cells was greatly improved (0.95, 0.88), and there was no significant difference compared to the control group. There were more complete tubular structures, which were significantly different from those in the simple Ox-LDL stimulation group (Figures 3(a) and 3(b)). At the same time, the Transwell results also reflected the same trend. In the antibody intervention group, the migration ability of the endothelial cells was greatly improved, which was not much different from that of the control group (0.75 ± 0.09), and there were significant differences compared to the Ox-LDL stimulation group (0.26 ± 0.07) (Figures 3(c) and 3(d)).

### 3.3. Specific Antibodies Inhibited the Inflammatory Response of the Endothelial Cells and the Expression of LOX-1

**Figure 4:**
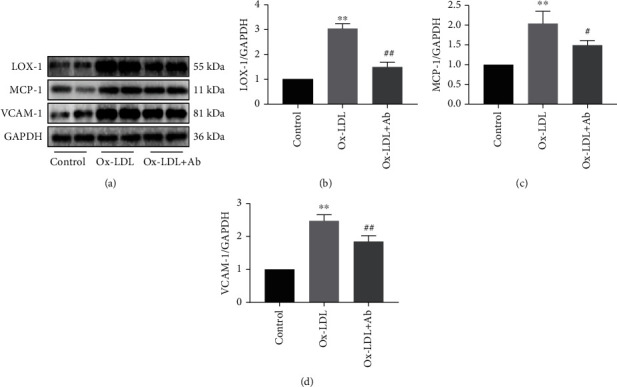
Specific antibodies inhibited the inflammatory response of the endothelial cells and the expression of LOX-1. (a–d) The expression of LOX-1, MCP-1, and VCAM-1 in the three groups and the corresponding statistical graphs. Values are expressed as mean ± SD. ^∗∗^*P* < 0.01 vs. the control group. ^#^*P* < 0.05 and ^##^*P* < 0.01 vs. the Ox-LDL group.

The effects of specific antibodies on the endothelial cells are depicted in Figure 4(a). The expression of LOX-1, MCP-1, and VCAM-1 in the endothelial cells in the Ox-LDL group was much higher than that in the control group. However, the expression of these proteins was lower in the Ox-LDL+Ab group than in the Ox-LDL group. From the statistical chart (Figures 4(b)–4(d)), we can see that Ox-LDL can activate endothelial inflammation; increase the expression of inflammatory molecules, MCP-1 and VCAM-1; and increase the expression of LOX-1. Compared with the control group, this effect was significantly different. After antibody intervention, this effect was obviously suppressed. These findings indicate that a small amount of LOX-1 is expressed in vascular endothelial cells under physiological conditions, but continuous Ox-LDL stimulation can increase the expression of LOX-1, thereby mediating more Ox-LDL deposition in endothelial cells. The above reaction caused an increase in the expression of inflammatory factors in endothelial cells and ultimately resulted in the destruction of the endothelial barrier and the death of endothelial cells. Immune intervention with an Ox-ApoB antibody can inhibit the expression of the endothelial cell inflammatory response induced by Ox-LDL and can inhibit the expression of LOX-1, protect endothelial cells, and avoid the destruction induced by Ox-LDL.

### 3.4. Specific Antibody Intervention Can Significantly Protect the Expression of Tight Junction Proteins in the Vascular Endothelial Cells

**Figure 5:**
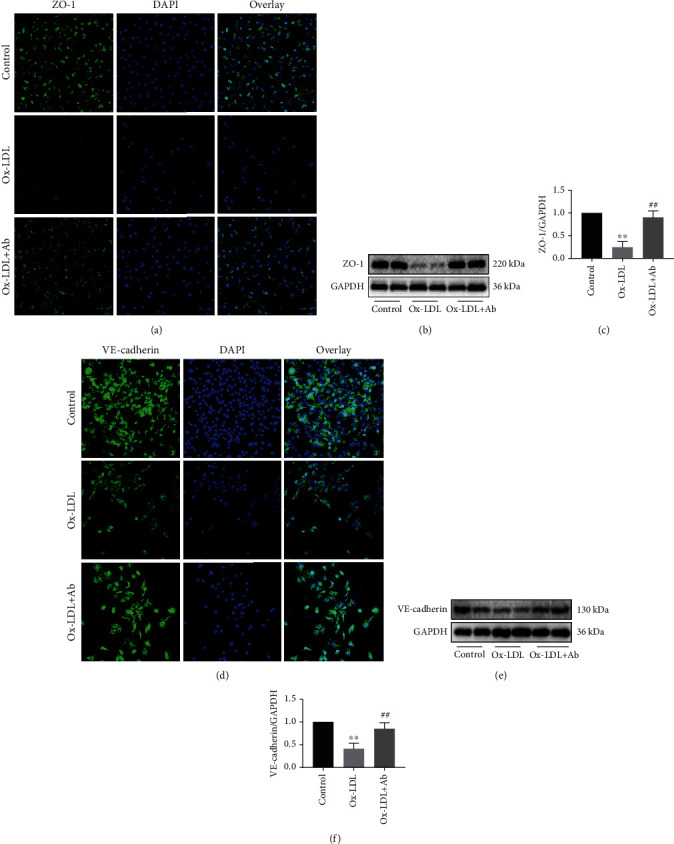
Specific antibody intervention can significantly protect the expression of tight junction proteins in the vascular endothelial cells. (a) Confocal laser shows the different expression levels of ZO-1 (200x) in the three groups, where blue represents the nucleus and green represents the expression of ZO-1. (b) Western blot shows the expression level of ZO-1 in the three groups. (c) Quantitative analysis statistics of the expression levels of ZO-1 in the three groups. (d) Confocal laser shows the different expression levels of VE-cadherin (200x) in the three groups, where blue represents the nucleus and green represents the expression of VE-cadherin. (e) Western blot shows the expression level of VE-cadherin in the three groups. (f) Quantitative analysis statistics of the expression levels of VE-cadherin in the three groups. Values are expressed as mean ± SD. ^∗∗^*P* < 0.01 vs. the control group. ^##^*P* < 0.01 vs. the Ox-LDL group.

An intact endothelium is essential for controlling the vascular permeability. The control of endothelial permeability involves the expression level of adhesion molecules and tight junction molecules [22]. The formation of adhesion junctions leads to tight junction assembly [23]. Therefore, we focused on ZO-1 and VE-cadherin, two adhesion junction proteins that are essential for the formation of endothelial cell-cell adhesion. The results that are shown in Figure 5(a) indicate that the expression of ZO-1 (green fluorescent) in endothelial cells in the Ox-LDL+Ab group was significantly increased compared to that in the Ox-LDL group. With an immune intervention with a specific antibody, the expression of tight junction proteins in the cells was remarkably improved compared with that in the Ox-LDL group (Figures 5(b) and 5(c)). Similarly, the expression of VE-cadherin also showed the same trend (Figures 5(d)–5(f)). Therefore, based on the above experimental results, we infer that the continuous stimulation of Ox-LDL has a serious effect on the tight junctions between vascular endothelial cells. The intervention with a specific antibody can maintain the tight junctions of vascular endothelial cells to a large extent and protect the integrity of the vascular endothelial barrier.

### 3.5. The Ox-ApoB Polypeptide Fragment Immune Intervention Significantly Inhibits the Progression of Atherosclerosis and Reduces the Area of the Necrotic Core in the Plaque

**Figure 6:**
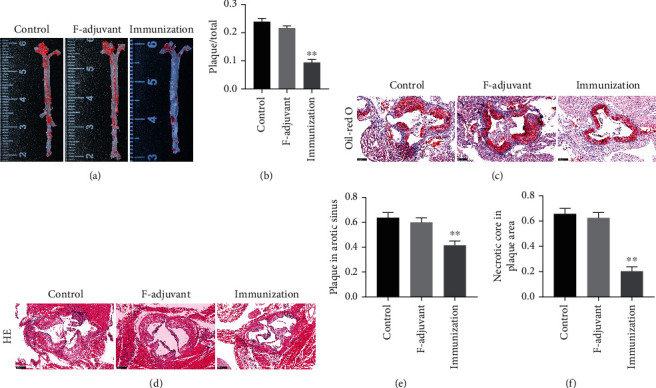
The Ox-ApoB peptide fragment immune intervention significantly inhibits the progression of atherosclerosis and reduces the area of the necrotic core in the plaque. (a, b) The inhibitory effect of Ox-ApoB fragments on atherosclerotic lesions in mice. The control group, the F-adjuvant group, and the immunization group are represented by aortic ORO staining images (a) and the percentage of the positive plaque area in the whole aorta (b). (c–f) Three representative images of the aortic valve annulus stained with ORO (100x) (c) and H&E (100x) (d). The statistical graph of the percentage of the plaque-positive area in the ORO staining in the aortic annulus (e) and the statistical graph of the percentage of the crystalline necrotic core area in the plaque in the H&E staining (f). Data are expressed as the mean ± SD. ^∗∗^*P* < 0.01 vs. the control group.

ApoE^−/−^ mice were fed a high-fat diet for 12 weeks with or without Ox-ApoB treatment. After staining the aorta with ORO, it was observed that the atherosclerotic lesions of ApoE^−/−^ mice in the immunization group were significantly inhibited compared with those of the control and F-adjuvant groups. The results of the study indicated that the immunization group (0.09 ± 0.01) showed a significant reduction in the extent of aortic atherosclerosis compared to the control group (0.24 ± 0.01), which was also reduced compared with the adjuvant group (0.22 ± 0.01) (Figures 6(a) and 6(b)). At the same time, for the quantitative analysis of the degree of atherosclerotic lesions, we judged by measuring the ORO-positive area in the aortic sinus. Compared with the plaque area of mice in the control group (0.64 ± 0.04), the plaque area of the aortic sinus of ApoE^−/−^ mice after immune intervention (0.42 ± 0.03) was significantly reduced (0.60 ± 0.04) (Figures 6(c) and 6(e)). In addition, for the quantitative analysis of the necrotic core area in the plaque, we evaluated the percentage of the area of the necrotic core area by H&E staining of the aortic sinus compared to the area of the plaque. The results showed that the crystalline necrotic core area in the aortic sinus plaque of ApoE^−/−^ mice after the intervention with Ox-ApoB (0.20 ± 0.04) was significantly reduced compared with that of the F-adjuvant alone (0.63 ± 0.04) (Figures 6(d) and 6(f)). The above data show that intervention with specific fragments of Ox-ApoB can inhibit the progression of atherosclerosis and can reduce the area of the necrotic core in the plaque, thus stabilizing the plaque.

### 3.6. Ox-ApoB Polypeptide Fragment Immune Intervention Reduces Inflammation and Macrophage Migration in Plaques

**Figure 7:**
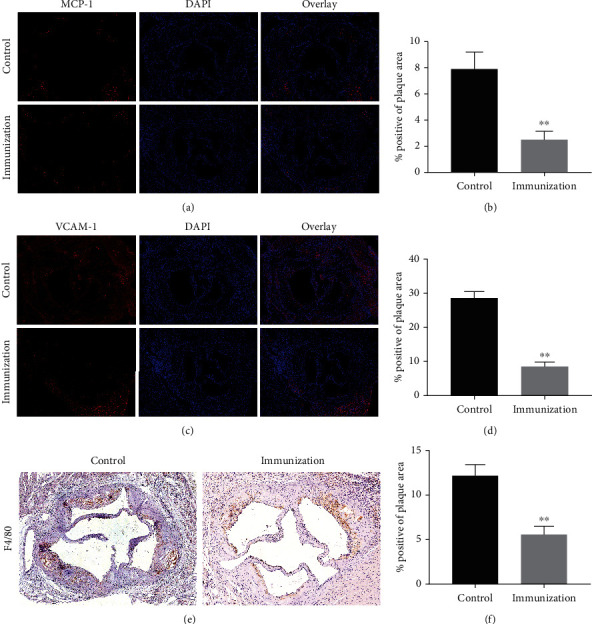
Ox-ApoB peptide fragment immune intervention reduces inflammation and macrophage migration in plaques. (a, b) Representative images of aortic root sections obtained from the control and immunization groups stained with MCP-1 (a). Nuclei were stained with DAPI (blue). The percentage of the plaque area that was occupied by MCP-1 is presented among the indicated groups (b). (c, d) Representative images of aortic root sections obtained from the control and immunization groups stained with VCAM-1 (c). Nuclei were stained with DAPI (blue). The percentage of the plaque area that was occupied by VCAM-1 is presented among the indicated groups (d). (e, f) Representative images of aortic root sections obtained from the control and immunization groups stained with F4/80 (e). The percentage of the plaque area that was occupied by F4/80 is presented among the indicated groups (f). Data are expressed as the mean ± SD. ^∗∗^*P* < 0.01.

The above results indicate that the progression of atherosclerosis in mice is significantly inhibited after intervention with Ox-ApoB fragments. The vascular endothelium serves as the first line of defense against adverse stimuli. Endothelial cells secrete MCP-1, VCAM-1, and other inflammatory factors under Ox-LDL stimulation, causing circulating mononuclear macrophages to continue to migrate under the endothelium, causing persistent inflammation in the plaque. To further verify the changes in inflammatory factors in the mouse atherosclerotic plaques after the intervention with an antibody, we carried out corresponding tests. First, we performed immunofluorescence to detect MCP-1 contained in the plaques in the three groups of aortic valve annuli. The results showed that the MCP-1 content in the plaque of the aortic sinus of ApoE^−/−^ mice after immune intervention (2.5 ± 0.66%) was significantly reduced compared to that in the control group (7.9 ± 1.3%) (Figures 7(a) and 7(b)). Similarly, the VCAM-1 immunofluorescence results also showed the same trends. The VCAM-1 content in the aortic sinus plaque of ApoE^−/−^ mice after Ox-ApoB intervention (8.5 ± 1.3%) was significantly reduced compared to that in the control group (28.6 ± 1.9%) (Figures 7(c) and 7(d)). In addition, we also tested the number of macrophages in the plaques. The results showed that the F4/80 content in the plaque of the aortic sinus of ApoE^−/−^ mice after Ox-ApoB intervention (5.6 ± 0.9%) was significantly reduced compared to that of the control group (12.2 ± 1.2%) (Figures 7(e) and 7(f)). These data show that intervention with Ox-ApoB significantly inhibits the inflammatory storm caused by various stimuli in the plaque, which can inhibit the progression of atherosclerosis and stabilize the plaque.

### 3.7. The Immune Intervention with the Ox-ApoB Polypeptide Fragment Protects the Vascular Endothelial Barrier Connection, Reduces the Expression of LOX-1 and Cell Apoptosis in the Plaque, and Increases the Clearance of Ox-LDL by Liver Macrophages

**Figure 8:**
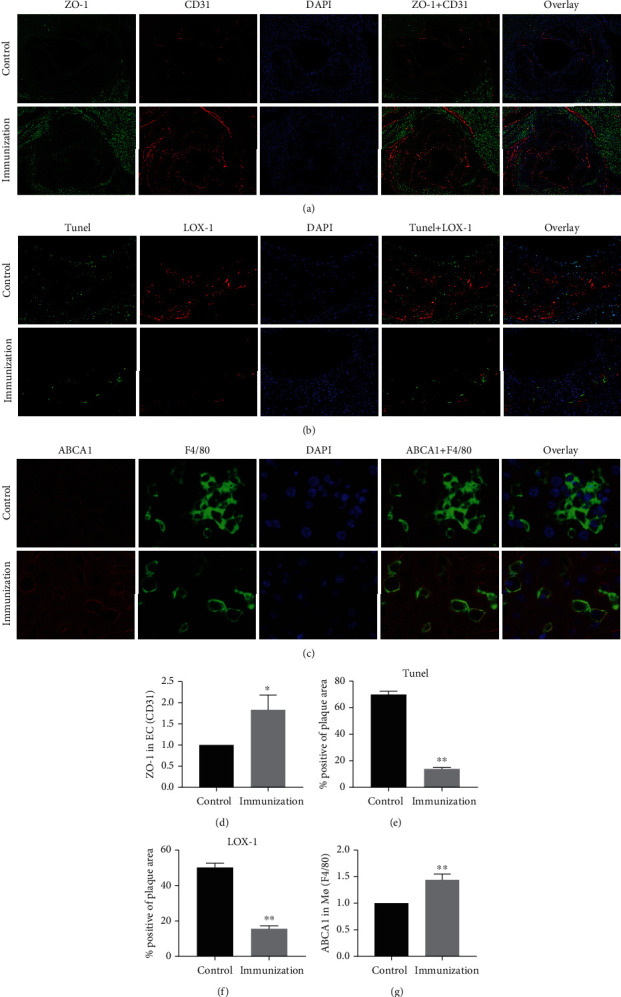
The immune intervention with the Ox-ApoB peptide fragment protects the vascular endothelial barrier connection, reduces the expression of LOX-1 and cell apoptosis in the plaque, and increases the clearance of Ox-LDL by liver macrophages. (a, d) Representative images of aortic root sections obtained from the control and immunization groups stained with ZO-1 and CD31 (a). Nuclei were stained with DAPI (blue). Compared with the control group (set as 1), the relative ratio of CD31 that was occupied by ZO-1 is presented among the indicated groups (d). (b, e, f) Representative images of aortic root sections obtained from the control and immunization groups stained with TUNEL and LOX-1 (b). Nuclei were stained with DAPI (blue). The percentage of the plaque area that was occupied by TUNEL and LOX-1 cells is presented among the indicated groups (e, f). (c, g) Representative images of liver sections obtained from the control and immunization groups stained with ABCA1 and F4/80 (c). Compared with the control group (set as 1), the relative ratio of F4/80 occupied by ABCA1 is presented among the indicated groups (g). Data are expressed as the mean ± SD. ^∗^*P* < 0.05, ^∗∗^*P* < 0.01.

To explore how Ox-ApoB fragments inhibit atherosclerotic lesions, we further explored their mechanism. First, the early occurrence and development of atherosclerosis is closely related to the imbalance of the endothelial junction barrier function, and vascular endothelial tight junction proteins are closely related to the integrity of the vascular endothelial barrier. We detected the endothelial cell markers CD31 and ZO-1 in plaques of the aortic roots using immunofluorescence staining (Figure 8(a)). We found that the expression of CD31 and ZO-1 in the immunization group (1.83 ± 0.4) was increased compared with that in the control group (set as 1) (Figure 8(d)). We further analyzed the expression of LOX-1 and the level of apoptosis in the plaques of the aortic roots using immunofluorescence staining (Figure 8(b)). Compared with the control group (69.8 ± 2.6% and 50.2 ± 2.4%), we found that the amount of TUNEL (as a positive marker of apoptosis) and LOX-1in the plaque was significantly reduced in the immunization group (13.8 ± 1.2% and 15.5 ± 1.8%) (Figures 8(e) and 8(f)). Additionally, we also detected the macrophage marker F4/80 and the expression of ABCA1 in the liver using immunofluorescence staining (Figure 8(c)). Compared with the control group (set as 1), the results indicated that the number of macrophages was reduced, while the expression of ABCA1 in the liver was increased (1.4 ± 0.1) (Figure 8(g)). From all these findings, we can conclude that the immune intervention with the Ox-ApoB polypeptide fragment protects the vascular endothelial barrier connection, reduces the expression of LOX-1 and cell apoptosis in the plaque, and increases the clearance of Ox-LDL by liver macrophages.

**Figure 9:**
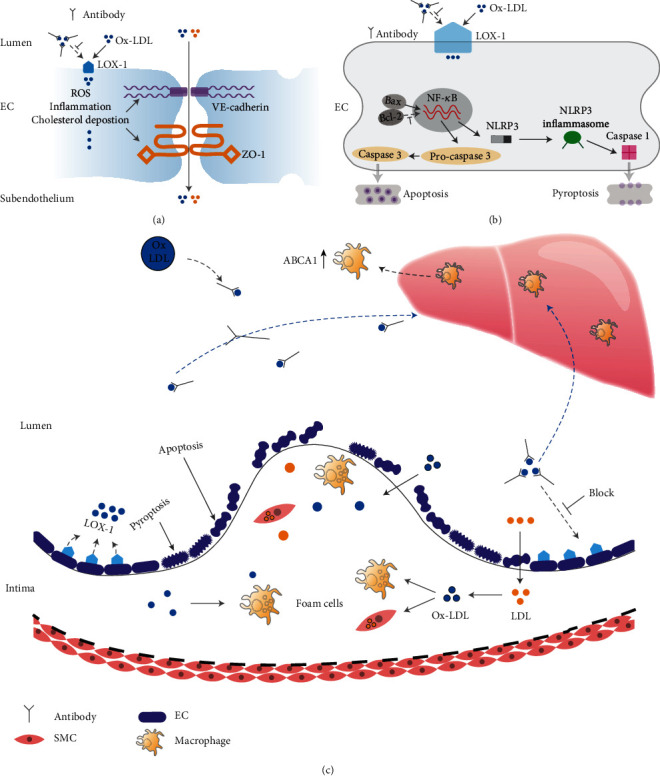
Schematic diagram of the experimental hypothesis.

Overall, in this study, we found that the LOX-1-mediated endothelial phagocytosis of Ox-LDL causes lipid deposition, oxidative stress, and inflammation in endothelial cells. These effects led to a decrease in the number of tight junctions, a decrease in the expression of adhesion junction proteins in endothelial cells, and an increase in lipid particle penetration and crevice penetration (Figure 9(a)). Meanwhile, this effect further activated the apoptosis and pyrolysis pathways of endothelial cells, which impaired endothelial function (Figure 9(b)). However, with the use of the Ox-ApoB fragments, this damage was significantly alleviated. Finally, redundant Ox-LDL was cleared by macrophages in the liver using the ABCA1 receptor (Figure 9(c)).

## 4. Discussion

In this study, our results show that Ox-LDL deposited on the blood vessel wall can be phagocytosed by endothelial cells and subendothelial macrophages after being oxidized to form Ox-LDL. This mechanism not only leads to the decrease in endothelial cell proliferation, migration, and the tube-forming function but also increases the expression of inflammatory and endothelial adhesion factors in endothelial cells, which can lead to a decrease in the expression of endothelial cell tight junction proteins. Through an immune intervention with specific Ox-ApoB fragments, we found that the damaging effects of Ox-LDL on the endothelial cell junction barrier were decreased, and the endothelial cell junction barrier was more protected, thereby inhibiting the progression of atherosclerosis.

Currently, acute cardiovascular events caused by atherosclerosis are still the leading cause of death in the world [24]. Vascular endothelial cell dysfunction caused by the deposition of oxidized lipids is considered to be the main cause of early atherosclerosis [12, 25]. Under physiological conditions, the vascular endothelium forms a monolayer cell structure through gap junctions, tight junctions, and adhesion junctions [26]. Under normal circumstances, endothelial cells allow some small molecules and fluids to pass through the cell and intercellular space, while albumin, LDL, etc. cannot easily penetrate the endothelial barrier in the circulation [27]. When there is the development of hyperlipidemia, hypertension, smoking, etc., the LDL in the circulation continuously form Ox-LDL through lipoxygenase and myeloperoxidase. In addition, studies have shown that lipid oxidation products (LOPs) are widely present in many fat-containing foods. They usually enter the gastrointestinal tract from dietary sources and/or are produced in the body [28]. Ox-LDL is deposited under the blood vessel wall and is phagocytosed by vascular endothelial cells, which in turn causes vascular endothelial cell dysfunction. An increasing number of studies have used Ox-LDL to stimulate HUVECs for in vitro studies of vascular endothelial dysfunction [29]. Compared with LDL, Ox-LDL plays a more critical role in the occurrence and development of atherosclerotic lesions [30]. Ox-LDL in the circulation accounts for approximately 10% of LDL in circulation. They can deposit in the vascular endothelial cells through LOX-1 receptors on the surface of endothelial cells and can cause a series of adverse consequences [31]. In this study, we found that the proliferation, migration, and tube formation of endothelial cells are inhibited after Ox-LDL stimulation, which is consistent with the conclusions of previous studies. In addition, stimulation with Ox-LDL increased the expression of inflammatory and adhesion factors in endothelial cells, and the expression of tight junction proteins between cells was significantly reduced. The in vivo experiments were also consistent with the in vitro experiments. Therefore, we believe that Ox-LDL stimulation can cause significant damage to vascular endothelial cells.

LOX-1 was originally found in bovine aortic endothelial cells and belongs to the scavenger receptor family. It is often expressed on the surface of vascular endothelial cells, smooth muscle cells, macrophages, etc. and can mediate the deposition of oxidized lipids in cells. Under physiological conditions, the expression of LOX-1 in endothelial cells is low, but its expression can be greatly increased under the stimulation of oxidized lipids in the circulation [17]. Unlike macrophages, endothelial cells cannot transport cholesterol outside of the cell through the cholesterol efflux pathways such as ABCA1/ABCG1 after phagocytosing Ox-LDL. Endothelial cells phagocytose Ox-LDL through LOX-1. The early elimination of oxidized lipids through the autophagy lysosomal pathway can barely maintain the stability of the endothelial cell environment, but continuous oxidized lipid deposition can cause intracellular oxidative stress and inflammation [32]. Endothelial cells are chemotactic and can induce circulating monocytes, macrophages, and lymphocytes to migrate under the inner membrane by releasing MCP-1 and VCAM-1 [33]. LOX-1 mediates Ox-LDL through NADPH oxidase and other pathways to induce an increase in ROS and endothelin (ET) expression. The deposited Ox-LDL also inhibits the activity of endothelial nitric oxide synthase (eNOS) and the production of the metabolite nitric oxide (NO), which initiates oxidative stress. The disturbance of the balance between endothelial contraction and relaxation accelerates the damage to the endothelial barrier [34]. The continuous inflammatory response causes the subendothelial elastic fiber membrane to be decomposed by matrix metalloproteinases, which intensifies the shedding of endothelial cells. These damages continue to accumulate and eventually lead to the death of endothelial cells and the destruction of the endothelial barrier. In addition to classic apoptosis, an increasing number of studies have shown that Ox-LDL can also induce pyrolysis of vascular endothelial cells [35]. In contrast to apoptosis and necrosis, endothelial pyrolysis releases a large number of inflammatory factors, which continuously aggravate the inflammatory cascade under the vascular intima. These factors are closely related to the continuous progression and rapid morbidity of atherosclerosis.

ApoB is the main component of low-density lipoproteins. The core pathogenic component of Ox-LDL is that oxidized ApoB is changed into an oxidation-specific epitope. Studies have shown that the capture of Ox-ApoB by vascular endothelial cells is the initial process that initiates and drives the atherosclerotic process [36]. In recent years, studies on ApoB-related vaccines have shown that the use of ApoB-derived vaccines can clearly inhibit the progression of atherosclerosis. However, the specific mechanisms related to this pathway still need to be studied in depth. Here, we used an Ox-ApoB antibody to coat Ox-LDL to observe the relevant effects and found that after the immune intervention, the expression of the tight junction proteins ZO-1 and VE-cadherin in mouse arterial endothelial cells was greatly improved. The secretion of macrophages, MCP-1, VCAM-1, and other factors, in the plaque were also significantly reduced. Therefore, we speculate that the use of specific Ox-ApoB fragments for immune intervention can reduce the deposition of Ox-LDL in endothelial cells can prevent the destruction of the endothelial junction barrier and thereby inhibit the progression of atherosclerosis.

Drug-based interventions play a key role in atherosclerotic lesions. Several recent clinical studies have shown that the combination of moderate-intensity statins and certain nonstatin lipid-lowering drugs have no additional clinical benefits and have more adverse reactions [37–39]. Treatment and prevention in the initial stage of atherosclerotic lesions by using an immune intervention is a promising research direction. In conclusion, our study explored the molecular mechanism of Ox-LDL-induced atherosclerosis in vitro and found that immune intervention with specific Ox-ApoB fragments can largely protect the endothelial cell connection barrier and thus prevents the apoptosis and necrosis of arterial endothelial cells caused by the stimulation of Ox-LDL and ultimately inhibits the progression of atherosclerosis.

## 5. Conclusions

The survival state and tight junctions of vascular endothelial cells are essential for maintaining the normal physiological functions of blood vessels. Phagocytosis and clearance of Ox-LDL by the LOX-1 receptor in vascular endothelial cells induced the dysfunction of vascular endothelial barrier and sustained intravascular inflammation. Ox-ApoB-PF immune intervention can reduce the accumulation of Ox-LDL through LOX-1 in endothelial cells, thereby maintaining the integrity of the vascular endothelial barrier and inhibiting the progression of atherosclerotic lesions. Our research provides preliminary research evidence for immune intervention to protect the survival and functional integrity of vascular endothelial cells.

## Data Availability

Data is included within the article.
